# Chronic Non-cirrhotic Portal Vein Thrombosis with Cavernous Transformation Secondary to Protein C and S Deficiency

**DOI:** 10.7759/cureus.7142

**Published:** 2020-02-29

**Authors:** Zainab Majid, Faryal Tahir, Taha Bin Arif, Jawad Ahmed

**Affiliations:** 1 Internal Medicine, Dow University of Health Sciences, Karachi, PAK

**Keywords:** hereditary thrombophilias, portal vein thrombosis, protein s deficiency, splenorenal shunt, splenectomy, protein c deficiency, cavernous transformation of the portal vein, anticoagulant therapy

## Abstract

Hereditary thrombophilia (HT), including the mutation of factor V gene and the deficiency of proteins C, protein S, or antithrombin, is a risk factor for portal vein thrombosis (PVT). PVT in acute cases is usually asymptomatic, whereas chronic cases mostly present as variceal bleeding and splenomegaly. However, cavernous transformation of the portal vein secondary to a long-standing PVT is very rare. Here we present a case of a 28-year-old female who was admitted with complaints of left upper abdominal pain and swelling for four to five years. Using laboratory and radiological examinations, a confirmatory diagnosis of cavernous transformation of a thrombosed portal vein due to protein C and S deficiency was made. The patient was managed through splenectomy with splenorenal shunting along with life-long prescription of anticoagulants.

## Introduction

The portal vein (PV) is formed at the confluence of the splenic vein (SV) and superior mesenteric vein (SMV) behind the pancreatic head. Portal vein thrombosis (PVT), a complete or partial obstruction to blood flow, is usually associated with a thrombus in the lumen of the PV [1]. PVT is classified into four categories: thrombosis limited to the PV beyond the junction of the SV and SMV; extension of thrombus into the SMV, but with patent mesenteric vessels; diffuse thrombosis of splanchnic venous system with large collateral vessels; and extensive splanchnic venous thrombosis with fine collateral vessels. Among other risk factors, hereditary thrombophilia (HT) is a known predisposition to PVT. It includes prothrombin gene G20210A or factor V Leiden mutations as well as deficiencies of one or more intrinsic anticoagulant proteins C and S, or antithrombin [2]. PVT does not affect the hepatic function unless the patient has an underlying liver pathology e.g. cirrhosis. In acute phases, diagnosis is usually missed opting to the asymptomatic nature of the disease. In chronic cases, 90% of the patients present with variceal bleeding, which occurs 4-12 years following extrahepatic PVT event. Moreover, splenomegaly is found in 75%-100% of the patients with chronic disease [3]. Opting to the increased use and advancement of non-invasive imaging modalities in the diagnostic evaluation of abdominal pain, acute portomesenteric venous obstruction has become a commonly recognized disorder [2]. The overall prognosis is good, with a 10-year survival of 75%, and an overall mortality rate of less than 10% [2].

Cavernous transformation of the portal vein (CTPV), a consequence of long-standing PVT, is a rare entity secondary to various etiologies and diverse clinical presentations [1]. It is characterized by the development and dilatation of multiple small collateral vessels in and around an occluded PV in the setting of portal hypertension (PHT), where ascites are less frequently seen [3]. CTPV has been found in non-cirrhotic and non-tumoral PVT with a healthy liver histology, yet the exact causes are unknown [1]. Patients usually present with gastroesophageal variceal bleeding and hematologic abnormalities due to enlargement of the spleen and the development of portosystemic collateral veins [4]. However, an accurate clinical diagnosis is very rare [1]. Abdominal ultrasonography (AUS), color Doppler ultrasonography, computed tomography (CT) angiography, and magnetic resonance imaging can be used to confirm the diagnosis of CTPV [4].

We present a case of a 28-year-old female who presented with complaints of left upper abdominal pain and swelling for four to five years. After a series of laboratory and radiological investigations, a diagnosis of cavernous transformation of a thrombosed PV secondary to the deficiency of proteins C and S was made. The patient underwent surgery for splenectomy along with splenorenal shunting (SRS) followed by a life-long prescription of anticoagulants.

## Case presentation

A 28-year-old female, with no known comorbidities, presented to the outpatient department with complaints of left upper abdominal pain and swelling for four to five years. The pain was spontaneous in onset, severe in intensity, radiating to the back and relieved on rest. It was not associated with food intake. The abdominal distension was gradually increasing in size. There was no history of fever or bleeding per rectum. Later she developed shortness of breath, nausea, and early satiety. Her past history was insignificant.

On examination, her blood pressure was 130/85 mm Hg, respiratory rate was 18 breaths per minute, and heart rate was 75 beats per minute. Abdominal examination revealed a soft, non-tender, grossly distended abdomen with centrally placed umbilicus. Splenic notch was palpable up to the umbilicus, firm and smooth in contour with regular margins. Bowel sounds were resonant. Neurological examination showed no focal neurological deficits with a Glasgow Coma Score of 15/15. Rest of the systemic examination was insignificant.

Laboratory evaluation on admission revealed hemoglobin (Hb) of 7.8 g/dL [normal (N)=12-15.5], hematocrit of 26% (N=36-46), mean corpuscular volume of 74 fL (N=80-100), mean corpuscular hemoglobin (MCH) of 22 pg (N=27-32), MCH concentration of 30 g/dL (N=31.5-34.5), total leukocyte count of 2.4x10^9^/L (N=4-10), and platelet count of 22x10^9^/L (N=150-400). Peripheral blood film depicted anisocytosis, poikilocytosis, hypochromia, and pancytopenia with teardrop cells. Considering the history of anemia, serum iron, red blood cell folate, and vitamin B12 were checked, which were found as 26 ug/dL (N=26-170), 1600.3 ng/mL (N=499-1504), and 294 pg/mL (N=206-678), respectively. The direct and indirect Coombs tests were negative. Serum electrolytes were also within normal ranges.

**Figure 1:**
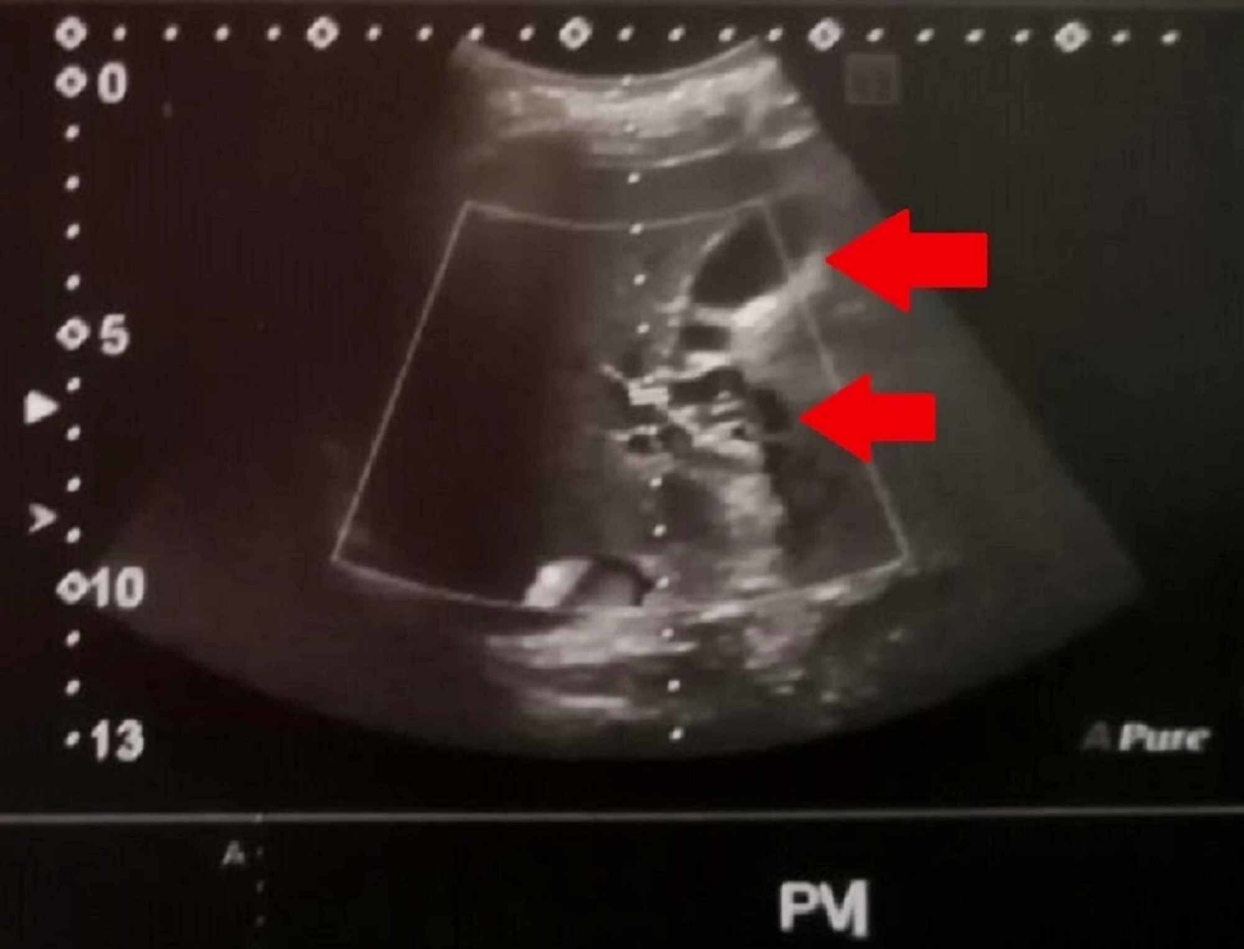
Doppler US showing CTPV (red arrows) US, ultrasound; CTPV, cavernous transformation of the portal vein

**Figure 2:**
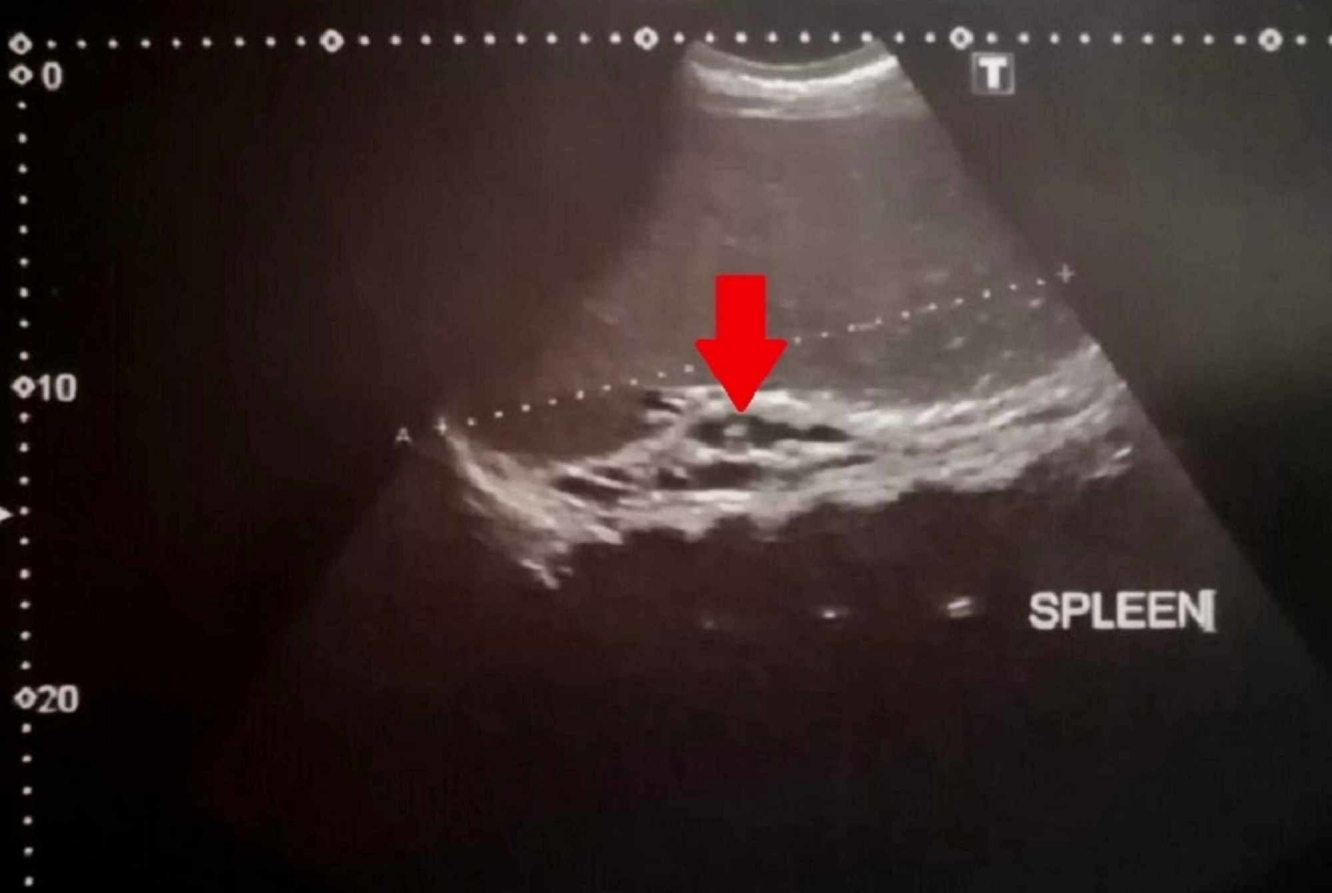
Doppler US showing dilated varices at splenic hilum (red arrow) US, ultrasound

The AUS report showed altered echotexture of the liver, grossly enlarged spleen measuring about 23 cm with dilated SV (1.2 cm) and mild ascites. In addition, chronic thrombosis with CTPV and few splenic varices at splenic hilum were observed in the Doppler study (Figures 1, 2).

Liver function tests (LFTs) showed total bilirubin of 0.9 mg/dL (N≤1.2), serum glutamic oxaloacetic transaminase of 20 U/L (N≤31), and alkaline phosphatase (ALP) of 26 IU/L (N=44-147), and gamma-glutamyl transferase of 16 U/L (N≤38). Viral markers were absent.

Keeping in view the abnormal Doppler study directed towards a procoagulant state, the patient was further considered for a detailed coagulation profile (Table 1).

**Table 1:** Coagulation profile of the patient PT, prothrombin time; INR, international normalized ratio; ATIII, antithrombin III; HCY, homocysteine; FVL, factor V Leiden

Test name	Patient’s value	Reference range
PT	12.1	11-12.5 s
INR	1.16	≤1.1
ATIII	100	80%-120%
Protein C	52	70%-130%
Protein S	56	71%-113%
HCY	10.68	4-15 µmol/L
FVL mutation	Absent	-

**Figure 3:**
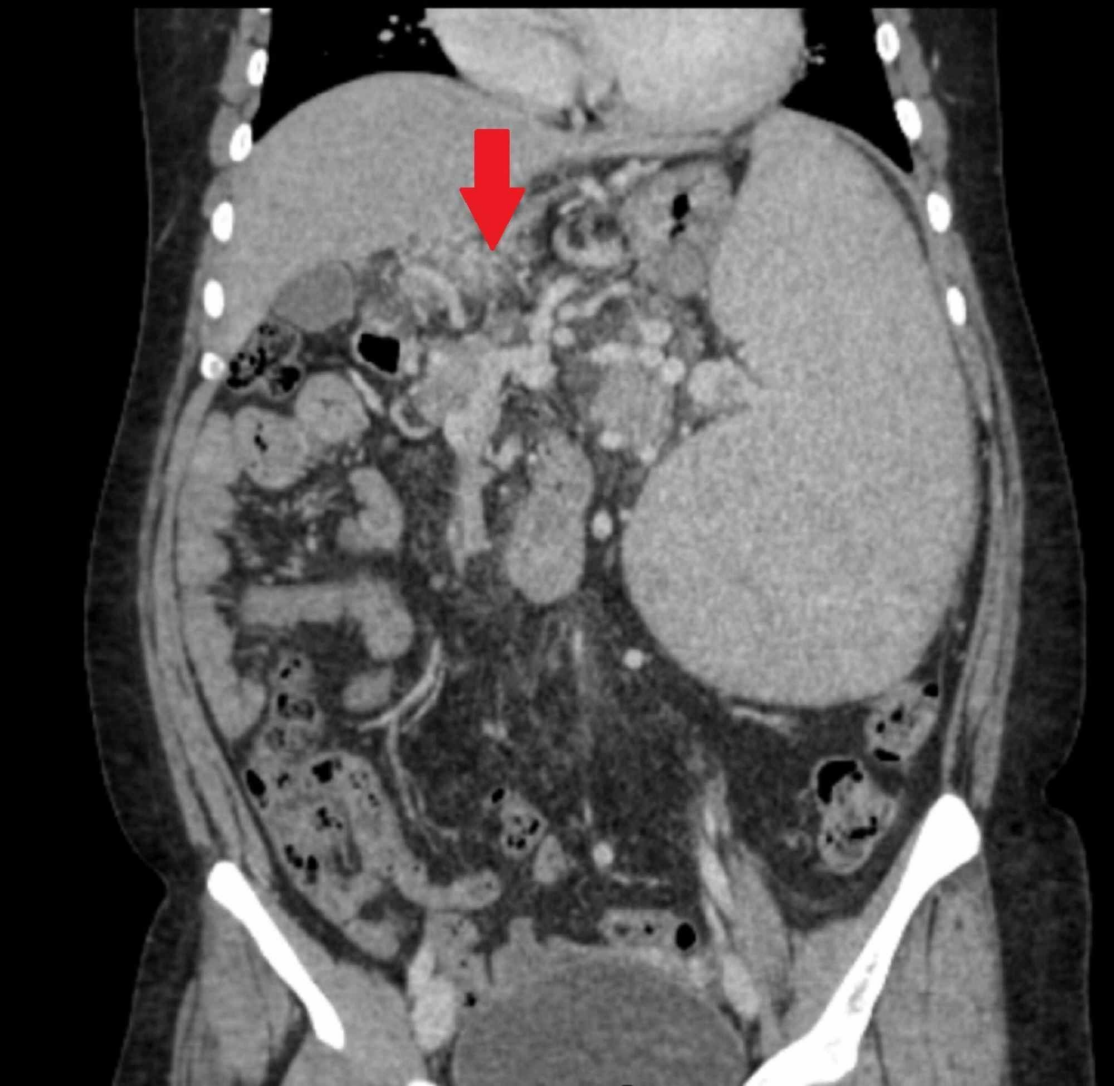
CT scan of abdomen (coronal view) showing collaterals or cavernoma formation around PV CT, computed tomography; PV, portal vein

**Figure 4:**
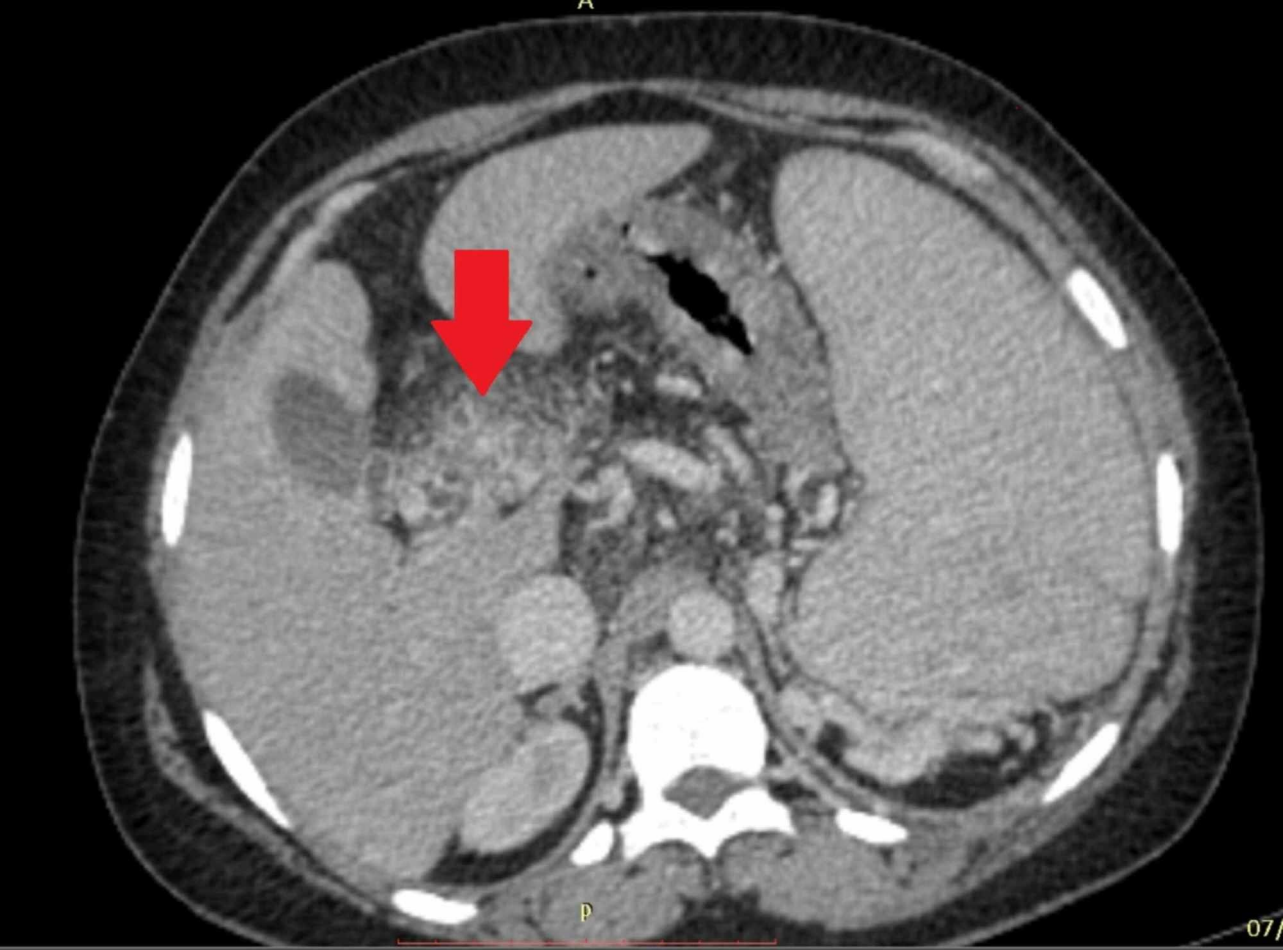
CT scan of abdomen (axial view) showing collaterals or cavernoma formation around PV CT, computed tomography; PV, portal vein

Multiple axial sections of CT scan of the abdomen and pelvis were carried out after non-ionic intravascular contrast enhancement. The liver with slight undulating margins and markedly attenuated caliber of PV up to the portosplenic confluence (6 mm approximately) with chronic intraluminal thrombus were observed. Multiple collaterals were formed at porta hepatis in keeping with the cavernous transformation (Figures 3, 4).

**Figure 5:**
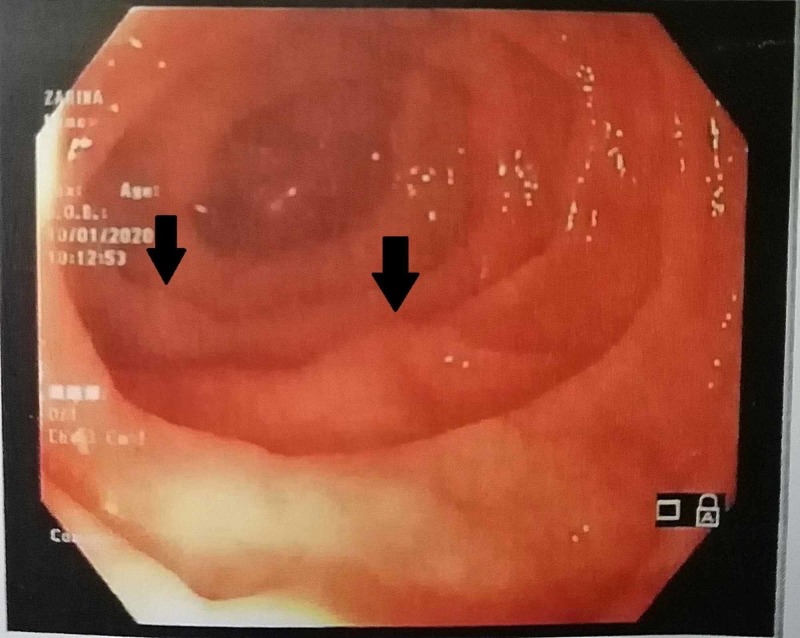
Upper GI endoscopy showing esophageal varices of grade 2 (black arrows) GI, gastrointestinal

This caused compression over the common bile duct at the porta hepatis resulting in mild prominence of intrahepatic biliary ducts. The spleen was markedly enlarged measuring approximately 21 cm. Extensive collateral vessels were seen at the splenic hilum in the peripancreatic and perirectal regions. A significant fat stranding was identified in the region of porta hepatis and in the root of mesentery with multiple prominent mesenteric lymph nodes. Upper gastrointestinal endoscopy revealed grade II esophageal varices (Figure 5). A final diagnosis of chronic non-cirrhotic PVT leading to massive splenomegaly with esophageal varices secondary to protein C and S deficiency was established.

As the thrombus in the PV was organized, thrombectomy could not be performed. A decision of splenectomy with end-to-side surgical shunting via SRS was made. Considering the patient was severely anemic and predisposed to several infections post-splenectomy, the Hb was initially improved to 10 g/dL along with vaccination against encapsulated organisms. Five pints of human blood was arranged before surgery to compensate for the blood loss during splenectomy.

Two weeks later, the patient underwent splenectomy with end-to-side SRS construction. The splenic artery was ligated, and the SV was clamped and divided. The remaining attachments of spleen were then divided, and spleen was removed en bloc. The SV was freed up to its confluence with the inferior mesenteric vein. The left renal vein (RV) was isolated through an incision over the renal hilum. The renal artery was clamped, and the SV was brought to the RV. An end-to-side splenorenal anastomosis was performed once an optimal length of the SV was obtained. Due to extensive bleeding, the patient was transfused with four pints of blood. Post-operatively, the patient was maintained on life-long anticoagulant therapy with frequent follow-ups.

## Discussion

Generally, veins have a wider diameter, lower pressure, and a slower blood flow in comparison with the arteries and, thereby, hold an increased risk for thrombosis. This is further explained by the Virchow’s triad, i.e. presence of endothelial injury, hypercoagulability, and blood stasis, and factors that contribute to the clot formation. Thrombosis in the PV is rarely observed and is reported in 1% of the population by a study on autopsies, while another study recorded prevalence of PVT in 3.7 per 100,000 individuals [5,6]. The first case of PVT was described back in 1868 by Balfour and Stewart, in which the patient developed portal ascites, splenomegaly, and variceal dilatation [7]. Several underlying pathologies leading to PVT as a consequence have been described, which include certain infections, malignancies, acute pancreatitis, use of oral contraceptives, pregnancy, hyperhomocysteinemia, and coagulopathies. Coagulopathies can either be inherited or acquired: the former includes protein C, protein S, and antithrombin deficiency and the latter comprises of myeloproliferative disorders, antiphospholipid syndrome, and paroxysmal nocturnal hemoglobinuria [8].

Protein C and protein S are naturally occurring anticoagulants, and their deficiencies have been described as an infrequent yet probable cause of PVT, the incidence of which is reported by a study as 11% [9]. Another study conducted by Fisher et al. in 2000 found combined protein C and S deficiencies to be 28%, in which the coagulation profiles in adult patients with PVT were assessed [10]. Protein C, a vitamin K-dependent zymogen, is activated by the binding of thrombin to thrombomodulin, with protein S acting as a cofactor for activated protein C (APC). APC, in turn, inactivates factor V and factor VIII, inhibiting the coagulation cascade; hence, their deficiency leaves the body vulnerable to abnormal clotting.

In nearly one-third of the cases of PVT, the cirrhotic liver is the main contributory factor and has even accounted for 6%-64% in autopsy studies [11,12]. The blood flow through the fibrosed hepatocytes causes increased resistance to blood flow and resultant blood stasis, thus fulfilling the Virchow’s effect and predisposing to clot formation. The presence of PVT without cirrhosis is rare, and is commonly associated with myeloproliferative neoplasms like polycythaemia rubra vera, essential thrombocythemia, and primary myelofibrosis [13]. Protein C and protein S deficiencies were reported as 0%-10% and 0%-30%, respectively, as risk factors for non-cirrhotic PVT [14]. Since our patient had normal LFTs and fibroscan without a significant history of risk factors for cirrhosis, other than the coagulopathy, she was classified as a case of non-cirrhotic PVT.

The clinical picture of PVT varies greatly depending on the acute or chronic presentation. The acute symptoms may include abdominal or lumbar pain, fever, new-onset ascites, and metabolic acidosis [14]. During later stages, the portosystemic shunting develops, leading to esophageal varices, rectal bleeding, and hematemesis along with symptoms of PHT [15]. Long-standing PVT may lead to the development of alternative dilatation of multiple venous channels around the thrombosed PV, thus bypassing the occlusion. This is known as portal cavernoma or CTPV. Although a rare complication, cavernoma tends to cause splenomegaly and subsequent variceal bleeding, and hematological abnormalities, usually in the setting of non-cirrhotic and non-malignant PVT [4].

Although the procedure of portocaval shunting has a higher reliability and improved outcomes in patients with PVT, we opted for the formation of SRS with splenectomy, since our patient had an established thrombus in the PV and anticoagulants had no role in its resolution. A study assessed the outcome of SRS in 28 patients with PHT and cirrhosis, where the former group reported no recurrent bleed and other complications [16].

## Conclusions

It is not necessary that concurrent protein C and S deficiencies should present early with a significant history of any thromboembolic disorder. As in our case, the patient presented in the adulthood without any history of a thromboembolic event. It is also important to keep an eye on the atypical presentation of such HT as they can present with a puzzling clinical picture. Our case presented with the non-specific symptoms that were atypical of a thromboembolic disorder. Yet, being in a resource-limited setting but with adequate investigations and prompt management, we were able to successfully manage our patient. As HT like protein C and S deficiencies are incurable and the patient requires a life-long therapy with anticoagulants, these individuals should be promptly assessed. Any delay in the screening for such abnormalities can lead to serious complications i.e. stroke, myocardial infarction, or even peripheral arterial occlusion. Therefore, siblings and parents of a diagnosed patient should also be screened for such inherited coagulation disorders.
